# Clinical Utility of Z‐Score Distribution Mapping in Epileptogenic Zone Localizations

**DOI:** 10.1002/cns.70876

**Published:** 2026-04-25

**Authors:** Jinkun Han, Penghu Wei, Sichang Chen, Yanfeng Yang, Yongzhi Shan, Guoguang Zhao

**Affiliations:** ^1^ Department of Neurosurgery Xuanwu Hospital Capital Medical University Beijing China

**Keywords:** epilepsy, positron emission tomography (PET), seizure onset zone, Z‐score distribution mapping

## Abstract

**Objectives:**

This study aimed to evaluate the clinical utility of positron emission tomography (PET)‐based Z‐score distribution mapping (Z‐map) in the noninvasive presurgical localization of seizure onset zones (SOZs), with a particular focus on regional differences in performance and methodological robustness.

**Methods:**

We analyzed a cohort of 120 patients with drug‐resistant epilepsy who underwent stereoelectroencephalography (SEEG) implantation between 2021 and 2024. Multimodal imaging data, including PET and structural MRI, were processed using FreeSurfer and 3D Slicer to reconstruct electrodes, segment gray matter, and generate Z‐maps. Hypometabolic regions were defined as the bottom 0.5% of Z‐scores. The classification performance of the Z‐map was validated against SEEG‐defined SOZs.

**Results:**

The Z‐map showed high classification performance in the frontal and parietal lobes (sensitivity: 0.74/0.72; κ: 0.68/0.65), but reduced effectiveness in the temporal and insular lobes (sensitivity: 0.41/0.65; κ: 0.44/0.47). The overall specificity (0.94) and negative predictive value (0.91) indicated strong exclusionary capability. Regional disparities were primarily attributed to anatomical complexity and technical limitations.

**Conclusion:**

Z‐map offers clinically valuable support for localizing SOZs in the frontal and parietal cortex and optimizing SEEG implantation, particularly in patients with inconclusive noninvasive findings. However, its limited sensitivity in temporal and insular regions and high dependency on preprocessing quality underscore the need for standardized pipelines and multimodal integration. Future research should focus on improving robustness and reproducibility to facilitate clinical translation.

## Graphical abstract

Z‐score distribution mapping (Z‐map) is a noninvasive technique for epilepsy surgery planning and provides a comprehensive evaluation of regional variations in the performance of Z‐maps, which is a complementary tool for optimizing stereoelectroencephalography (SEEG) electrode implantation, especially in patients with inconclusive imaging findings or complex epileptogenic networks.

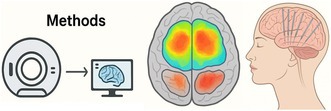

## 1 Introduction

Epilepsy is a common chronic neurological disorder characterized by recurrent seizures caused by abnormal electrical discharges in the brain. According to the World Health Organization, approximately 6.38 per 1000 people worldwide are affected [1]. While antiepileptic drugs remain the mainstay of treatment, around 30%–40% of patients show insufficient response and are classified as having drug‐resistant epilepsy [2]. For these individuals, surgical intervention has become a key therapeutic strategy. However, the effectiveness of epilepsy surgery largely depends on the accuracy of presurgical localization of the epileptogenic zone (EZ), which remains challenging, especially in cases involving frontal lobe epilepsy, insular epilepsy, or MRI‐negative findings [3, 4]. Improving localization precision is thus essential to optimize surgical outcomes and reduce recurrence.

As a functional imaging technique, positron emission tomography (PET) allows detection of cerebral hypometabolism and contributes to the assessment of EZs. However, studies have shown that even healthy individuals may exhibit physiological hypometabolism at rest [5], which can lead to false‐positive interpretations in standard visual assessments. To address this issue, we developed a quantitative analysis approach‐Z‐map which compares individual PET data to healthy controls to reduce physiological variability and highlight regions of abnormal metabolism.

Although the Z‐map offers potential advantages, its clinical application in presurgical epilepsy evaluation has been rarely reported. This study aims to explore its value as a reference tool in presurgical localization, particularly by assessing its regional performance characteristics and feasibility in clinical settings.

## 2 Methods

We analyzed patients diagnosed with drug‐resistant epilepsy at the Xuanwu Hospital Epilepsy Center who underwent stereoelectroencephalography (SEEG) implantation between 2021 and 2024. After screening, 120 patients with complete and analyzable datasets, including SEEG recordings, electrode localization data, 3D T1‐weighted MRI, and PET imaging, were included in the study.

### 2.1 Image Acquisition and Partial Volume Correction

Structural MRI was performed at Xuanwu Hospital using a 3.0 T GE Premier scanner (GE Healthcare, Waukesha, WI). High‐resolution T1‐weighted images were obtained with a magnetization‐prepared rapid gradient‐echo (MPRAGE) sequence using the following parameters: isotropic voxel size = 1.0 mm^3^, field of view = 256 × 256 mm^2^, repetition time = 2476 ms, echo time = 2.7 ms, and inversion time = 900 ms.

PET imaging was conducted using a United Imaging PET scanner (United Imaging Medical Technology Co. Ltd., Shanghai, China) with ^18^F‐fluorodeoxyglucose (^18^F‐FDG). Patients fasted for at least 6 h and had no seizures within 12 h before scanning. The ^18^F‐FDG tracer was administered intravenously at 3.7 MBq per kilogram of body weight. Image acquisition began 30 min postinjection and lasted 30–40 min. Reconstructed images had a voxel size of 1.82 × 1.82 × 2.78 mm^3^, a matrix size of 192 × 192 × 89, slice thickness of 2.4 mm, and isotropic spatial resolution of 5 mm.

### 2.2 SEEG Electrode Implantation

SEEG electrode implantation was performed using the ROSA robotic guidance system (Medtech, Montpellier, France), guided by preoperative 3D contrast‐enhanced T1‐weighted MRI data and planned with the Alcis stereotactic system (Besançon, France). Each depth electrode included 5–18 cylindrical contacts, with a diameter of 0.8 mm, a length of 2 mm, and 1.5 mm spacing between contacts.

### 2.3 Postoperative Electrode Reconstruction and Gray Matter Localization

Electrode reconstruction followed a standardized workflow. Preoperative T1‐MPRAGE images were processed using FreeSurfer (v6.0.0, https://surfer.nmr.mgh.harvard.edu/fswiki/FreeSurferWiki) for whole‐brain structural segmentation. The resulting segmentation masks were imported into 3D Slicer (v5.2.2, http://www.slicer.org/), where postoperative thin‐slice CT and preoperative MPRAGE DICOM data were coregistered using six‐degree‐of‐freedom rigid registration. Electrode contacts visible on CT were manually marked in the fused space, allowing for spatial visualization and coordinate extraction through Slicer's 3D navigation module.

For gray matter localization, the Destrieux atlas [6], embedded within FreeSurfer, was applied. This parcellation scheme divides the cortex into 74 bilateral regions based on gyral and sulcal curvature features. Electrode coordinates from postoperative CT were mapped to Destrieux regions using spatial analysis in MATLAB (2022a, https://ww2.mathworks.cn/). A contact was considered to reside in gray matter only if more than 50% of its voxels fell within a single Destrieux region. Subsequent SEEG interpretation was restricted to these verified contacts.

### 2.4 Analysis Process of the Z‐Map

The structural T1‐weighted MRI image was interpolated to isotropic voxel dimensions to establish a reference coordinate system. A skull‐stripping mask was generated using an adaptive threshold segmentation algorithm (intensity coefficient = 0.4) combined with 3D Gaussian smoothing (*σ* = 2 mm). PET data were acquired in 3D list mode with MRI‐based attenuation correction (Dixon two‐point method, 18 s). Image reconstruction employed the ordered subsets expectation maximization algorithm (8 iterations, 32 subsets), followed by Gaussian smoothing (FWHM = 4.5 mm) using a 3.0 mm kernel. Multimodal registration was conducted via a 12‐degree‐of‐freedom affine transformation model with normalized cross‐correlation (NCC > 0.85). Partial volume correction was applied using the van Cittert iterative method (http://www.turkupetcenter.net/tpcclib‐doc/md_install.html).

Brain tissue segmentation was performed using the “recon‐all” pipeline in FreeSurfer. PET images were converted to standardized uptake value ratios (SUVR) using the cerebellum as a reference region. Cortical surface mapping was conducted using the CBM method to reduce intersubject spatial variability by projecting PET data onto the fsaverage template surface (Gaussian kernel, FWHM = 20 mm). Sampling occurred at 50% cortical depth along the surface normal, preserving the native 3D vertex topology.

A 3D metabolic template was constructed from 23 healthy controls, and all statistical analyses were conducted in RStudio (https://www.rstudio.com/products/rstudio/download/). Global mean normalization was applied to minimize intersubject metabolic variability. Local deviations were quantified by vertex‐wise Z‐score calculation: Z = (individual value−group mean)/group standard deviation. Surface‐level processing was completed using the freesurferformats package (https://github.com/dfsp‐spirit/freesurferformats), and final Z‐maps were visualized on the individual's cortical surface using FreeView for metabolic‐anatomical fusion display.

### 2.5 Selection of Low‐Metabolism Regions and Definition of Statistical Indicators

For each patient, a Z‐map was generated and analyzed on a vertex‐wise basis. The 0.5% of vertices with the lowest Z‐scores were selected, and the corresponding anatomical regions were identified using the Destrieux atlas, with left and right hemispheres distinguished. These regions were defined as low‐metabolism areas.

SEEG electrode contacts localized to gray matter regions were used as reference for classification. Two binary categorizations were established: (1) seizure onset zone (SOZ) versus non‐SOZ regions, based on SEEG interpretation; and (2) low‐metabolism versus nonlow‐metabolism regions, based on Z‐map analysis. These classifications were cross‐compared to calculate the following six performance metrics:

Sensitivity (True Positive Rate, TPR): the proportion of actual SOZ regions correctly identified as hypometabolic by the Z‐map.

Specificity (True Negative Rate, TNR): the proportion of non‐SOZ regions correctly identified as nonhypometabolic.

Positive Predictive Value (PPV): the proportion of low‐metabolism regions that correspond to SOZ regions.

Negative Predictive Value (NPV): the proportion of nonlow‐metabolism regions that correspond to non‐SOZ regions.

Accuracy (ACC): the overall proportion of consistent classifications between the Z‐map and SEEG.

Kappa Coefficient: the agreement between Z‐map and SEEG classifications after adjusting for chance.

## 3 Results

### 3.1 Relevant Statistics on Brain Region Classification

Table 1 summarizes the demographic characteristics of the 120 patients with drug‐resistant epilepsy who underwent SEEG implantation, along with the 23 healthy controls included for Z‐map template construction. The median age was 24 years (range: 4–64) in the patient group and 31 years (range: 22–45) in the control group, with a wider age distribution observed among patients. Based on the Destrieux atlas, 74 ipsilateral gray matter regions of interest (ROIs) were anatomically classified. Among these, 28 were located in the frontal lobe, 11 in the parietal lobe, 14 in the temporal lobe, 13 in the occipital lobe, 8 in the insular cortex, and 1 region remained unclassified.

**TABLE 1:** Clinical characteristics of patients and HC.

Characteristics	Patients	HC
Quantity	120	23
Age (median)	24 (4–64)	31 (22–45)
Gender		
Male	52	9
Female	68	14
Dominant hemisphere (right)	117	23

A total of 1471 SEEG electrode contacts were identified as localized within gray matter ROIs. Region‐specific distribution showed that 559 contacts were located in the frontal lobe, 494 in the temporal lobe, 166 in the parietal lobe, and 186 in the insular cortex. According to the anatomical classification of the ROIs associated with these contacts, a total of 1260 brain region instances were included in the classification analysis—499 from the frontal lobe, 397 from the temporal lobe, 140 from the parietal lobe, and 163 from the insular cortex.

Table 2 presents the classification performance of the Z‐map in identifying SOZs across different brain regions. The results demonstrate notable regional variability in classification efficacy. Due to the limited number of occipital lobe regions available for analysis, their results were excluded from statistical reporting owing to potential estimation bias. Across all brain regions combined, the Z‐map showed strong specificity (TNR = 0.94) and NPV (0.91), indicating a reliable ability to exclude non‐SOZ regions. Overall classification accuracy was 0.87, and the Kappa coefficient reached 0.58, suggesting moderate agreement with SEEG‐based SOZ localization. The highest classification performance was observed in the frontal lobe, with sensitivity and specificity both reaching 0.74 and 0.94, respectively, and a Kappa value of 0.68. Similarly, the parietal lobe demonstrated balanced and favorable performance (sensitivity = 0.72; specificity = 0.93; Kappa = 0.65).

**TABLE 2:** Classification performance of Z‐map.

Category	TPR	TNR	PPV	NPV	ACC	Kappa
Total	0.62	0.94	0.70	0.91	0.87	0.58
Frontal lobe	0.74	0.94	0.74	0.94	0.90	0.68
Parietal lobe	0.72	0.93	0.72	0.93	0.89	0.65
Temporal lobe	0.41	0.96	0.69	0.88	0.86	0.44
Insular lobe	0.65	0.85	0.56	0.89	0.80	0.47
Left hemisphere	0.63	0.93	0.65	0.92	0.87	0.57
Right hemisphere	0.60	0.94	0.75	0.90	0.87	0.59

In contrast, the Z‐map showed limited sensitivity in the temporal and insular lobes. For the temporal lobe, although specificity was excellent (0.96), the sensitivity was markedly lower (0.41), indicating that while the method effectively excludes non‐SOZs in this region, it has difficulty correctly identifying actual SOZs. In the insular cortex, performance across all indicators was comparatively lower than in the frontal and parietal regions, with a sensitivity of 0.65 and a Kappa of 0.47. These findings suggest that the Z‐map performs reliably in frontal and parietal lobe SOZ classification, with consistent accuracy and balanced identification of positive and negative cases. However, its application in the temporal and insular lobes remains limited, particularly due to reduced sensitivity, reflecting challenges in localizing EZs within these anatomically complex areas.

### 3.2 Representative Case Demonstrations

**FIGURE 1:**
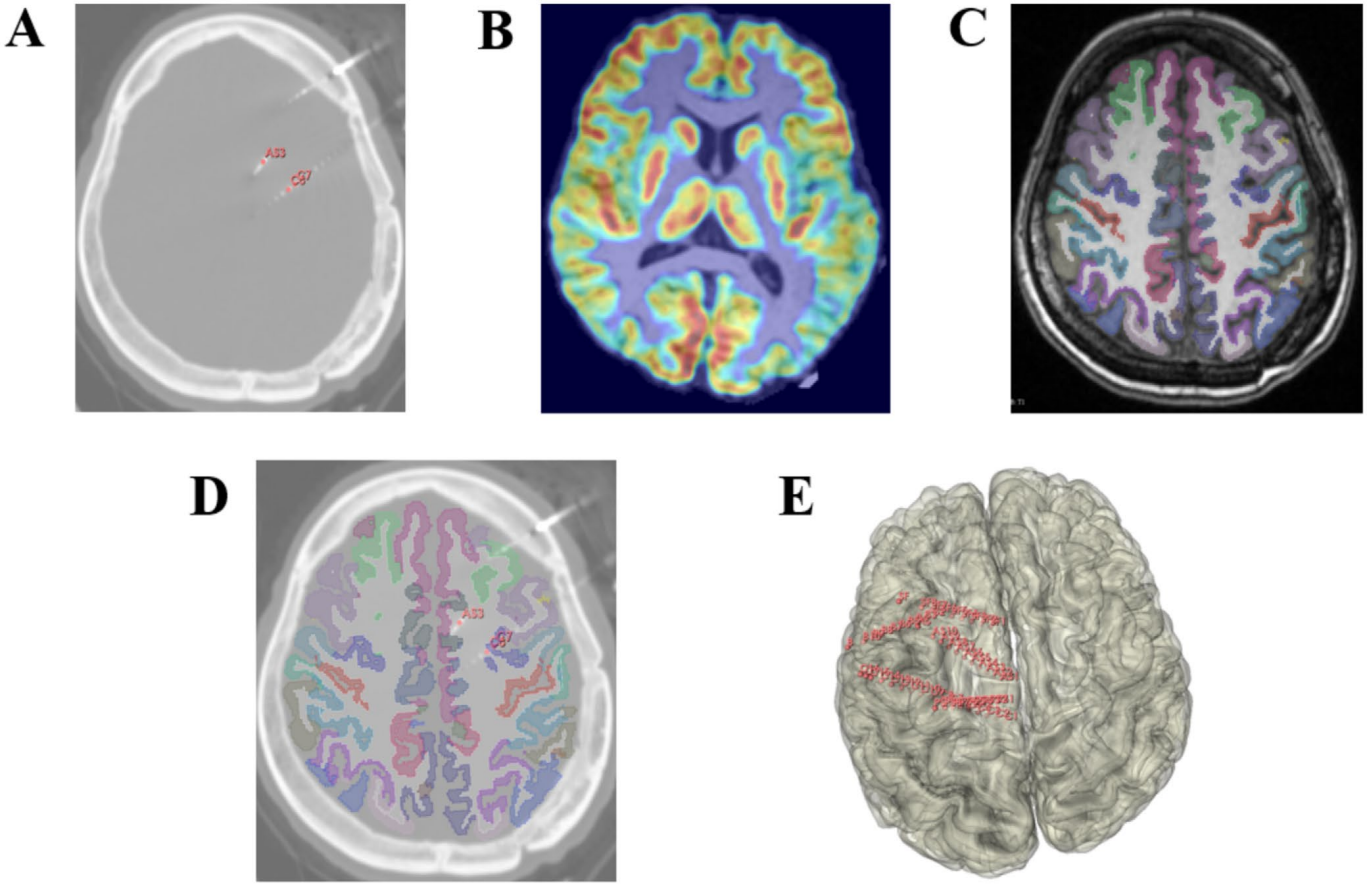
(A), (B), (C) Multimodality image processing based on single patient; (D) Positional relationship between electrode contacts and gray matter ROIs in axial images based on single patient; (E) Display of electrode contacts based on single patient in 3D space.

In this patient, the preoperative PET scan revealed hypometabolism in the left frontotemporal and central regions (Figure 1B). SEEG electrode placement is shown in Figure 1A, with labeled contact points. Figure 1C displays cortical segmentation results derived from FreeSurfer. In Figure 1D, electrode contact C7 is shown to be located in the precentral sulcus (Sprecentral‐sup‐part), as confirmed by voxel‐wise MATLAB analysis indicating that over 50% of the contact volume resided within that ROI. SEEG recordings at this site captured the onset of epileptic discharges. Figure 1E presents a 3D reconstruction of the electrode array in the patient's brain space.

**FIGURE 2:**
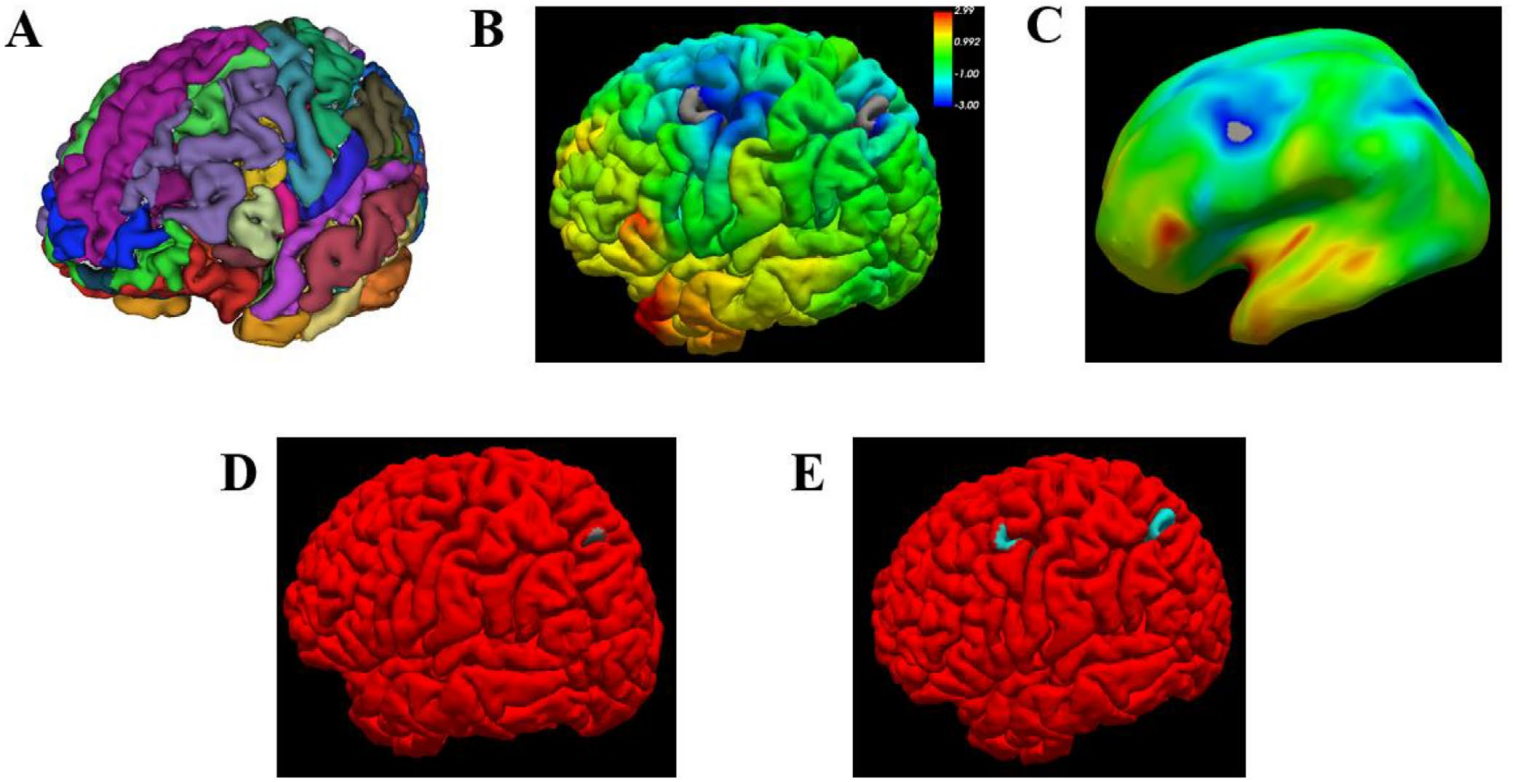
(A) Three‐dimensional segmentation results of Destrieux brain atlas in individual space; (B) The projection results of Z‐map on individual brain gyri; (C) Projection results of Z‐map on individual inflated surface; (D) Flexible adjustment of Z‐map threshold according to clinical needs; (E) Project after screening the Z values of different vertices by threshold.

Figure 2 illustrates the process of brain region reconstruction and 3D surface‐based PET analysis in this patient. As shown in Figure 2A, individual gray matter segmentation was performed based on the Destrieux atlas, generating a detailed anatomical model. In Figure 2B, PET‐derived Z‐map data were projected onto the native cortical surface using FreeView, revealing that multiple gyri in the left central and occipital regions had Z‐scores below −3.00, indicating metabolic values more than three standard deviations lower than those of healthy controls.

The display threshold in FreeView can be freely adjusted, allowing for rapid clinical interpretation. While the native cortical surface offers a clear view of gyral anatomy, it is less effective for visualizing sulcal regions. For example, in Figure 2D, after lowering the threshold to visualize more subtle hypometabolism, only the occipital lobe appears to fall below the threshold, even though the region with the lowest Z‐score is actually located in the central sulcus. To resolve this, visualization on the inflated surface (Figure 2C) was employed. Further threshold adjustment (Figure 2E) revealed that, among all cortical regions, only the central sulcus maintained Z‐scores below the set threshold, indicating it as the area of most pronounced hypometabolism. Notably, this region was subsequently targeted for surgical resection via awake craniotomy. The patient remained seizure‐free at 6‐month postoperative follow‐up, providing clinical support for the Z‐map‐based localization of the epileptogenic focus.

## 4 Discussion

Successful epilepsy surgery depends critically on accurate localization of the EZ [7]. However, presurgical evaluation for drug‐resistant epilepsy remains challenging. Noninvasive imaging modalities such as MRI and PET provide valuable information about epileptogenic networks, but their limited spatial resolution and specificity often lead to imprecise localization. SEEG remains the important standard for EZ delineation, yet its invasiveness, high cost, and surgical risks underscore the need for improved presurgical strategies to minimize unnecessary electrode implantation and enhance localization efficiency [8]. In this context, our study assessed the classification performance of PET‐based Z‐map analysis using SEEG‐defined SOZs as the reference standard. We aimed to clarify the regional applicability and limitations of this method, thereby establishing its role as a potential adjunct in presurgical localization.

### 4.1 Regional Heterogeneity in Z‐Map Classification Performance

The results revealed substantial regional variability in the performance of Z‐map‐based SOZ classification. While the method demonstrated high specificity (0.94) and NPV (0.91), its moderate sensitivity (0.62) suggests its primary utility lies in excluding nonepileptogenic regions rather than serving as a standalone diagnostic tool.

Strong classification concordance was observed in the frontal and parietal lobes, with kappa values of 0.68 and 0.65, and sensitivities of 0.74 and 0.72, respectively. In contrast, performance in the temporal and insular lobes was notably lower, with kappa values of 0.44 and 0.47. The temporal lobe's low sensitivity (0.41) likely reflects technical challenges in detecting deep‐seated epileptogenic activity in anatomically complex regions.

Bilateral comparisons showed similar overall agreement (kappa: left = 0.57; right = 0.59), but the right hemisphere exhibited a higher PPV (0.75 vs. 0.65), possibly due to metabolic lateralization or cohort‐related sampling differences.

Overall, Z‐map provides meaningful reference value for SOZ identification in the frontal and parietal lobes, but its limited sensitivity in temporal and insular regions highlights the need for integration with other presurgical localization modalities in these areas.

### 4.2 Potential Mechanisms Underlying Regional Performance Differences

The superior classification performance of the Z‐map in frontal and parietal regions may be attributed to several anatomical and electrophysiological factors. Structurally, the frontal and parietal cortex exhibits relatively uniform gyral architecture [9] and thicker gray matter, which together reduce partial volume effects in PET signal acquisition [10]. These features improve spatial alignment between hypometabolic regions and SEEG‐defined epileptogenic activity. Electrophysiologically, frontal lobe epilepsy is often characterized by focal cortical hyperexcitability, where suppression of metabolism aligns with high‐frequency oscillatory activity, forming distinct metabolic‐electrophysiological signatures [11]. Additionally, the parietal lobe features well‐segregated functional zones, particularly in the sensorimotor cortex [12], which limits cross‐regional metabolic overlap and enhances classification precision.

By contrast, the temporal lobe's low sensitivity may stem from both anatomical limitations and sampling bias. Mesial temporal EZs are frequently located in deep structures such as the hippocampus and amygdala [5], which are not captured by the neocortical surface‐based Z‐map. The Destrieux atlas also excludes these subcortical regions, preventing visualization of PET abnormalities associated with hippocampal discharges, even when detected by SEEG. Furthermore, patients selected for SEEG often present with complex epileptogenic networks and inconclusive noninvasive imaging, introducing cohort bias that may reduce Z‐map performance in this region. In the insular cortex, reduced classification efficacy may result from its small volume, which increases susceptibility to partial volume effects and attenuated metabolic signal. In addition, insular electrode contacts frequently span multiple small, densely packed ROIs. This cross‐boundary distribution can cause genuine SOZ contacts to be mislabeled or excluded from analysis, thereby lowering sensitivity.

Our findings revealed a clear spatial correlation between frontal and parietal regions of low Z‐scores and SEEG‐defined SOZs, supporting the potential role of focal hypometabolism as a surrogate marker of epileptogenicity. This observation is consistent with findings reported by Courtney et al. [13]. In contrast, the markedly lower sensitivity in the temporal lobe observed in our study appears largely attributable to methodological constraints, particularly the inability of surface‐based Z‐maps to capture metabolic abnormalities in deep mesial structures such as the hippocampus and amygdala. These limitations underscore the need for further optimization of Z‐map algorithms when evaluating limbic system involvement.

### 4.3 Advantages and Limitations of Z‐Map

The Z‐map offers a novel quantitative approach for noninvasive presurgical localization in epilepsy. Its primary advantage lies in providing a standardized framework for visualizing cortical metabolic deviations, enabling objective screening of frontal and parietal EZs. When validated against SEEG, the Z‐map demonstrated robust classification performance in these regions, supporting its role as an adjunct tool for refining SEEG implantation strategies—particularly in cases with discordant imaging findings or suspected foci near eloquent cortex.

A key limitation of the Z‐map is its high sensitivity to variations in technical processing. Small errors in skull stripping or image registration can propagate through the pipeline, leading to false‐positive or false‐negative localizations. This dependency on idealized preprocessing conditions reduces reliability in real‐world clinical settings, where scanner variability and anatomical heterogeneity are common. To enable broader clinical translation, future work should focus on developing standardized preprocessing workflows and automated quality control mechanisms. Such refinements are essential to reduce operator dependence and enhance the reproducibility and robustness of Z‐map‐based localization.

## 5 Conclusion

Our findings validate that PET‐derived Z‐map provides clinically significant reference value in noninvasive localization of SOZs within frontal and parietal regions, while offering strategic guidance for optimizing SEEG electrode implantation coverage. However, future investigations must prioritize developing robust preprocessing pipelines and registration algorithms to minimize operational variability at critical process, thereby enabling reliable clinical translation.

## Funding

This work was supported by Beijing Municipal Administration of Hospitals, ZLRK202319.

## Ethics Statement

This study was supported by Beijing Hospitals Authority Clinical Medicine Development of Special Funding (“YANG FAN” Plan) Clinical Technology Innovation Project (ZLRK202319), and was conducted in accordance with the principles outlined in the Declaration of Helsinki. Ethics approval for the research protocol was obtained from the Institutional Review Board of Xuanwu Hospital, Capital Medical University (Approval ID: Clinical Research Review (2024)266‐001). Patient confidentiality was strictly maintained, and all data were anonymized before analysis. The authors affirm that there are no conflicts of interest related to this work and that the study was carried out following all institutional and national ethics guidelines for research involving human participants.

## Conflicts of Interest

The authors declare no conflicts of interest.

## Data Availability

The data that support the findings of this study are available from the corresponding author upon reasonable request.

## References

[1] O. E. Zubareva , A. V. Dyomina , A. A. Kovalenko , et al., “Beneficial Effects of Probiotic *Bifidobacterium longum* in a Lithium‐Pilocarpine Model of Temporal Lobe Epilepsy in Rats,” International Journal of Molecular Sciences 24, no. 9 (2023): 8451. 37176158 10.3390/ijms24098451 PMC10179354

[2] R. Bresnahan , R. A. Hill , and J. Wang , “Perampanel Add‐On for Drug‐Resistant Focal Epilepsy,” Cochrane Database of Systematic Reviews 4, no. 4 (2023): Cd010961. 37059702 10.1002/14651858.CD010961.pub2 PMC10103545

[3] I. Oane , A. Barborica , and I. Mindruta , “Ictal Semiology in Temporo‐Frontal Epilepsy: A Systematic Review and Meta‐Analysis,” Epileptic Disorders 27 (2024): 171–186. 39724402 10.1002/epd2.20328 PMC12067355

[4] N. Cameron , L. Fry , J. L. Kabangu , et al., “Using Pre‐Surgical Suspicion to Guide Insula Implantation Strategy,” Heliyon 9, no. 7 (2023): e18284. 37539155 10.1016/j.heliyon.2023.e18284 PMC10395527

[5] M. E. Van Engelen , S. C. J. Verfaillie , A. Dols , et al., “Altered Brain Metabolism in Frontotemporal Dementia and Psychiatric Disorders: Involvement of the Anterior Cingulate Cortex,” EJNMMI Research 13, no. 1 (2023): 71. 37493827 10.1186/s13550-023-01020-2 PMC10371967

[6] C. Destrieux , B. Fischl , A. Dale , and E. Halgren , “Automatic Parcellation of Human Cortical Gyri and Sulci Using Standard Anatomical Nomenclature,” NeuroImage 53, no. 1 (2010): 1–15. 20547229 10.1016/j.neuroimage.2010.06.010 PMC2937159

[7] L. Yao , N. Cheng , A. Q. Chen , et al., “Advances in Neuroimaging and Multiple Post‐Processing Techniques for Epileptogenic Zone Detection of Drug‐Resistant Epilepsy,” Journal of Magnetic Resonance Imaging 60, no. 6 (2024): 2309–2331. 38014782 10.1002/jmri.29157

[8] K. Kobayashi and A. Ikeda , “Ictal Semiology Important for Electrode Implantation and Interpretation of Stereoelectroencephalography,” Neurologia Medico‐Chirurgica (Tokyo) 64, no. 6 (2024): 215–221. 10.2176/jns-nmc.2023-0265 PMC11230871 38719581

[9] D. Kiss‐Bodolay , A. Al Awadhi , K. O. Lovblad , et al., “The Fork Sign: A New Cortical Landmark in the Human Brain,” Brain Communications 6, no. 6 (2024): fcae398. 39564127 10.1093/braincomms/fcae398 PMC11576098

[10] M. Ibaraki , K. Matsubara , Y. Shinohara , et al., “Brain Partial Volume Correction With Point Spreading Function Reconstruction in High‐Resolution Digital PET: Comparison With an MR‐Based Method in FDG Imaging,” Annals of Nuclear Medicine 36, no. 8 (2022): 717–727. 35616808 10.1007/s12149-022-01753-5 PMC9304042

[11] B. Zhao , A. Mcgonigal , W. Hu , et al., “Interictal HFO and FDG‐PET Correlation Predicts Surgical Outcome Following SEEG,” Epilepsia 64, no. 3 (2023): 667–677. 36510851 10.1111/epi.17485

[12] D. Carius , E. Kaminski , M. Clauss , and P. Ragert , “Dynamic Alterations in Cortical Activation During Motor Adaptation in Table Tennis Using Whole‐Brain fNIRS,” Scientific Reports 15, no. 1 (2025): 10399. 40140446 10.1038/s41598-025-94699-3 PMC11947452

[13] M. R. Courtney , A. Antonic‐Baker , Z. Chen , et al., “Association of Localizing (18)F‐FDG‐PET Hypometabolism and Outcome Following Epilepsy Surgery: Systematic Review and Meta‐Analysis,” Neurology 102, no. 9 (2024): e209304. 38626375 10.1212/WNL.0000000000209304

